# Thermoelectric Photosensor Based on Ultrathin Single-Crystalline Si Films †

**DOI:** 10.3390/s19061427

**Published:** 2019-03-22

**Authors:** Gustavo Gonçalves Dalkiranis, Pablo Ferrando-Villalba, Aitor Lopeandia-Fernández, Llibertat Abad-Muñoz, Javier Rodríguez-Viejo

**Affiliations:** 1 Grup de Nanomaterials i Microsistemes, Departament de Física, Universitat Autònoma de Barcelona, Bellaterra, 08193 Barcelona, Spain; pablo.ferrandovillalba@imec.be (P.F.-V.); aitor.lopeandia@uab.cat (A.L.-F.); javier.rodriguez@uab.cat (J.R.-V.); 2 Instituto de Microelectrónica de Barcelona—Centre Nacional de Microelectrònica, Campus UAB, Bellaterra, 08193 Barcelona, Spain; llibertat.abad@imb-cnm.csic.es

**Keywords:** photosensor, thermoelectric effect, microsensor

## Abstract

Ultrathin Si films have a reduced thermal conductivity in comparison to Si bulk due to phonon scattering at the surfaces. Furthermore, the small thickness guarantees a reduced thermal mass (in the µJ/K range), which opens up the possibility of developing thermal sensors with a high sensitivity. Based on these premises, a thermoelectric (TE) microsensor based on ultrathin suspended Si films was developed and used as a thermal photosensor. The photoresponse of the device was evaluated with an argon laser (λ = 457 nm) with a variable power ranging from 0 to 10 mW in air at atmospheric pressure, with laser diodes at 406 nm, 520 nm and 638 nm wavelengths, and fixed powers in high vacuum conditions. The responsivity per unit area, response time (τ) and detectivity (D*) of the device were determined in air at ambient pressure, being 2.6 × 10^7^ V/Wm^2^, ~4.3 ms and $2.86\times 10^{7}\;{cmHz}^{\left(1/2\right)}W^{-1}$, respectively. Temperature differences up to 30 K between the central hot region and the Si frame were achieved during open-circuit voltage measurements, with and without laser diodes. During illumination, the photogeneration of carriers caused a slight reduction of the Seebeck coefficient, which did not significantly change the sensitivity of the device. Moreover, the measurements performed with light beam chopped at different frequencies evidenced the quick response of the device. The temperature gradients applied to the thermoelectric Si legs were corrected using finite element modeling (FEM) due to the non-flat temperature profile generated during the experiments.

## 1. Introduction

Photosensors are sensors that can detect light (electromagnetic waves). Depending on the desired spectral sensitivity, different physical principles should be considered. Among the sensors using semiconductors as active materials, this study highlights those working directly from [1,2,3,4]:

(i) Photoelectric or photovoltaic effects, where photons have energies over the semiconductor band gap;
(ii) Thermal-based absorption sensors, where the photon energies require the generation of mid-gap states to provide energy to the phonon bath.

Thermal detectors, either bolometers or thermoelectric-based (thermopiles), have a broad spectral range of detection and rely on the temperature increase produced by the absorbed radiation. Among the wide family of thermal absorption photosensors, those based on the thermoelectric (TE) principle do not require an external current or voltage source, and therefore may offer extremely low energy detection limits depending on the design. In general, thermal detection is preferred over photon detection for applications involving small excitation energies. Strategies to enhance the efficiency of thermoelectric detectors entail the improvement of the thermoelectric performance of the materials by maximizing its power factor, S^2^σ, which is the product of the square of the Seebeck coefficient and the electrical conductivity. In addition, low heat capacities per unit area and low thermal conductance are required to attain high temperature differences upon radiation absorption. A higher ΔT implies higher sensitivities. One method of achieving this goal is to use ultrathin highly doped Si layers as active elements of the n,p thermoelectric materials. It has been previously shown that reducing the dimensionality of Si can boost its thermoelectric performance reaching for small diameter Si nanowires, with ZT = S^2^σTk^-1^ values close to 1 at 300 K due to their reduced thermal conductivity close or even below the amorphous limit [5,6]. Similar results can be obtained using ultrathin Si membranes where enhanced scattering at the membrane surface dramatically affects the lattice thermal conductivity [7,8]. Additionally, appropriate designs and materials can be used to boost absorption at the sensing/absorbing regions. The simplicity of these types of devices enable their miniaturization. Previous work has shown that this strategy can be applied satisfactorily to fabricate Si-based compatible thermoelectric microsensors complementary metal-oxide-semiconductor (CMOS)-compatible microgenerators as energy harvesters for low-power applications [9,10,11,12].

This study presents a thermoelectric microsensor that relies on an array of ultrathin single-crystalline n- and p-type thermoelectric legs to generate the output voltage. The device was microfabricated from a silicon-on-insulator (SOI) wafer where a central silicon free-standing membrane (0.25 mm^2^ area) was connected and supported to the bulk silicon frame with 40, 100 nm thick thermoelectric silicon legs (20 doped with B and 20 doped with P) [11].

## 2. Materials and Methods

**Figure 1:**
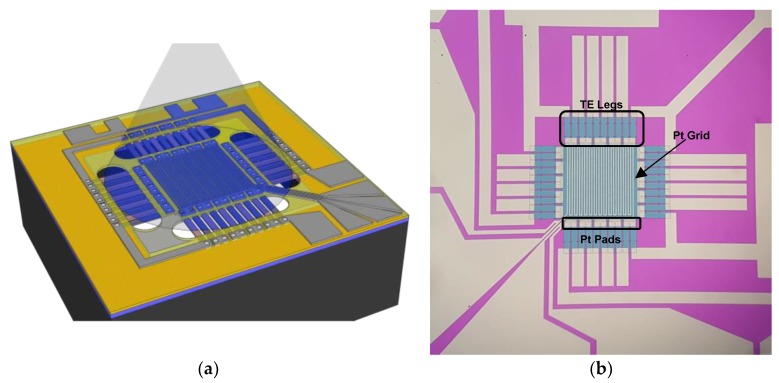
(**a**) Schematic of the device showing the illuminated area. (**b**) Optical image of the device presenting thermoelectric (TE) legs, Pt grid and Pt pads.

As shown in Figure 1, the device consisted of an array of 40 n,p thermocouples connected thermally in parallel and electrically in series between a central region (hot side) and a cold region (outside the Si frame). For a given temperature difference, the output voltage of the device was the sum of the voltage induced in every thermocouple. The device was equipped with electrical actuators to permit a full thermoelectric characterization (Figure 1). In the center of the device, a platinum grid was used as a heater and/or thermometer, which permits byJoule effect to increase the temperature and to establish temperature differences between hot (center of the device) and cold (frame of the device) sides. In parallel, the electrical properties of the thermoelectric legs can be evaluated. To use the platinum grid as a thermometer, the temperature coefficient of resistance (TCR) was obtained through a low-current (thus negligible self-heating) measurement of its electrical resistance at different temperatures close to 300 K. The thermoelectric legs had a doping level of 6.5 × 10^18^ cm^-3^ for p-type Si and 2 × 10^19^ cm^-3^ for n-type Si, and were connected electrically in series, leading to an internal resistance of ~62 KΩ. The doping levels of the n- and p-type legs were achieved through a sequential ion implantation (P and B) and rapid thermal annealing to recrystallize and activate the dopant atoms. The Si thermoelectric legs were 100 nm thick and showed a reduction in the thermal conductivity from 150 W/mK for bulk silicon at room temperature down to 60 W/mK, as reported elsewhere. The reduction in thermal conductivity was due to phonon scattering at the surfaces/interfaces [7,8,13,14,15]. On the contrary, the Seebeck coefficient remained close to expected values for bulk Si with similar doping levels (~5 × 10^19^ at/cm^3^) [5,6]. The thermocouples supported the SiN_X_ membrane that hosted the platinum (Pt) grid in the center of the device, which mechanically connected the membrane to the silicon frame. The membrane-based configuration of the device guaranteed a reduced thermal mass for sensing areas in the square millimeter range, making it ideal as a thermal photosensor. In the resulting device, the thermal sensing part of 0.5 mm^2^ was thermally isolated from the supporting Si frame (6 mm^2^) with a thermal conductance of 52 µW/K under high vacuum conditions. Finite element modeling (FEM) was required to consider the non-flat temperature profiles of the membrane during the characterization experiments. The microfabrication processes have been fully described by Perez-Marin et al. [11].

The measurements were performed on the responsivity to light absorption illuminating the sensing zone with an Ar^+^ laser (λ = 457 nm) at different powers up to 10 mW in ambient conditions. While the sensing zone was illuminated, the temperature difference and Seebeck voltage generated were measured. The absorbance of the central multilayer stack (non-optimized) membrane was around 52% at 457 nm, as shown in the transmittance and reflectance spectra in the Supplementary Materials (Figure S1). In addition, using the platinum grid as a heat source, open voltage circuit measurements were carried out in a vacuum with and without illumination by using 406 nm, 520 nm and 638 nm laser diodes operating at 2.94 mW, 3.8 mW and 4.2 mW, respectively. To heat only the central zone, the laser beam was collimated by an objective lens, as schematically shown in Figure 1a.

## 3. Results

**Figure 2:**
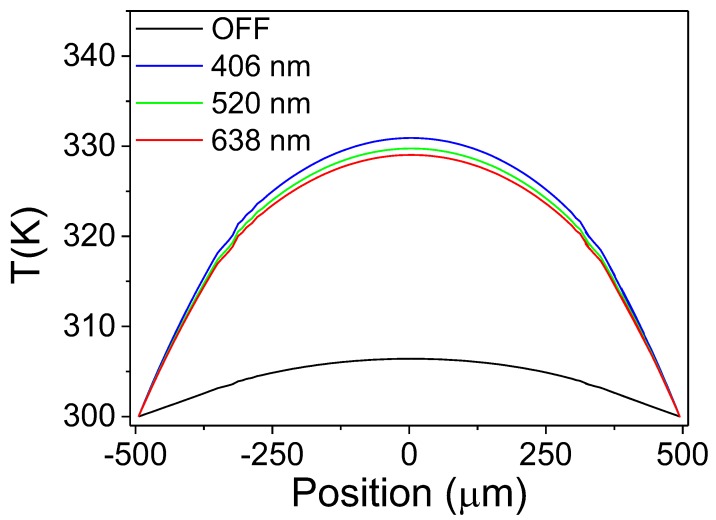
Temperature as a function of the position from the center of the device simulated by finite element modeling (FEM).

Due to the non-flat temperature profiles generated during the experiments, FEM corrections were required. The correction consisted of establishing a relationship between the average temperature measured by the platinum grid (heat source and/or thermometer) and the average temperature at the hot junction (Pt pads) of the thermoelectric Si legs (see Figure 1b). Based on this analysis a correction factor was calculated and used to obtain the real temperature gradient applied on the thermoelectric Si legs. For the illuminated device, the heating caused by the light must be also considered in the simulations due to local changes in the temperature distribution. Therefore, the power of the light was considered as an additional heat source and the absorption coefficient of each element of the device was used to estimate the amount of heat absorbed from the laser beam. Figure 2 shows the temperature profile in each case, in dark conditions and when the device was illuminated by the different laser diodes at 406 nm, 520 nm and 638 nm operating at 2.94 mW, 3.8 mW and 4.2 mW, respectively. It is clear that the temperature increased and the temperature profiles changed due to the incident radiation. It is also important to note that the shape of the temperature distribution changed with the different wavelengths used, mainly due to the variation of the absorption coefficients of the different wavelengths.

The correction of the temperature drop in the thermoelectric legs was carried out as follows: First, the mean temperature of the platinum grid $\left(\bar{T_{G}}\right)$ was calculated using the FEM results. Then, the mean temperature in the platinum pads $\left(\bar{T_{P}}\right)$ at the hot junction was evaluated using the FEM results, thus allowing the calculation of the correction factor as follows:

(1) $$f=\frac{\bar{T_{G}}-T_{S}}{\bar{T_{P}}-T_{S}}$$

with $T_{S}$ being the substrate temperature. The values of the correction factor for the illuminated device changed as a function of the temperature rise generated by the platinum grid, from 1.45 to 1.61 for the different temperature gradients. In dark conditions, this factor remained almost constant at 1.71. Thus, the real temperature difference applied to the thermoelectric legs was obtained by dividing the temperature difference measured experimentally by the correction factor.

**Figure 3:**
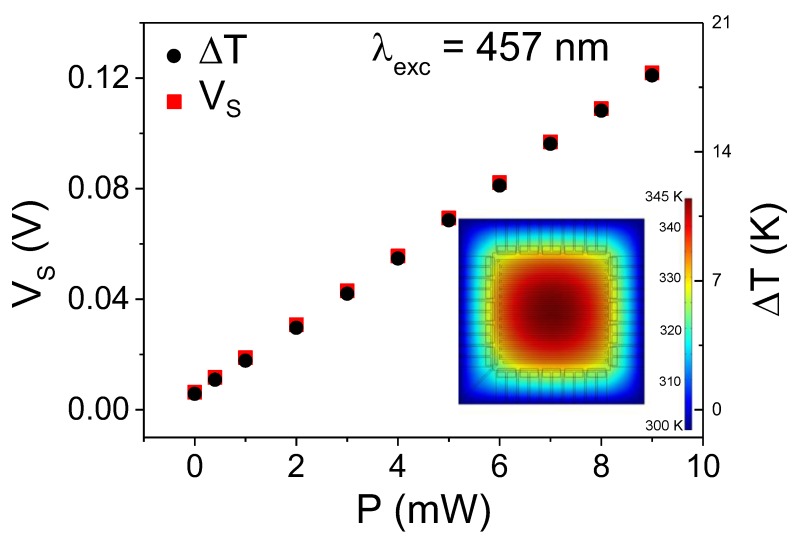
Plot of the open-circuit voltage at and temperature difference as a function of laser power. The inset plot shows the temperature profiles simulated by FEM.

Figure 3 shows the voltage generated in the TE legs as a function of the laser power. The linear increase of the Seebeck voltage, V_S_, was due to the temperature difference induced by the laser beam between the central region and the silicon frame (black circles). The responsivity, R_S_, of the TE sensor, defined as the ratio between the output voltage and the absorbed power [16,17,18], was determined by the slope in Figure 3. R_S_ amounted to 13 V/W and yielded an outstanding value of 2.6 × 10^7^ V/Wm^2^ in air, considering the small area of the device.

**Figure 4:**
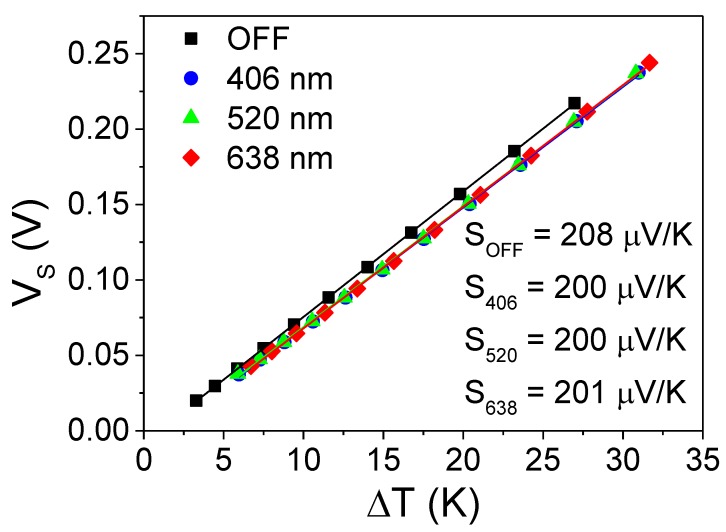
Plot of the Seebeck open-circuit voltage as a function of the temperature difference. The device is illuminated at 406 nm, 520 nm and 638 nm and without light.

Figure 4 shows the variation of the Seebeck open-circuit voltage as a function of the temperature difference between the thermoelectric legs, both under the illumination of 406 nm, 520 nm and 638 nm laser diodes and in dark conditions. The temperature difference was imposed by producing Joule heating in the platinum grid in the range of 0.24 mW to 2.37 mW. The incident light at 406 nm induced an additional temperature rise of ~2.7 K above the small temperature differences produced by Joule heating, which generated a voltage of ~17 mV as seen by the offset of the blue line in Figure 4. The other incident wavelengths, 520 nm and 638 nm, produced temperature rises of ~2.6 K and ~3.4 K, respectively. This generated an increase in the output voltage roughly similar to the light at 406 nm, as indicated by the offset of the green and red lines in Figure 4. This contribution decreased as the temperature gradient increased in agreement with a mechanism in which photogenerated carriers contribute to increase the electrical conductivity at the expense of reducing the Seebeck coefficient [19,20,21,22,23]. The small change in the Seebeck coefficient from 208 µV/K to 200 µV/K did not significantly modify the sensitivity of the device, enabling its use as a thermal detector in a broad range of wavelength radiation. The difference between the light power and the absorption coefficient of silicon attributed to each wavelength generated a similar number of electrical carriers, which led to a comparable reduction in the Seebeck coefficient.

**Figure 5:**
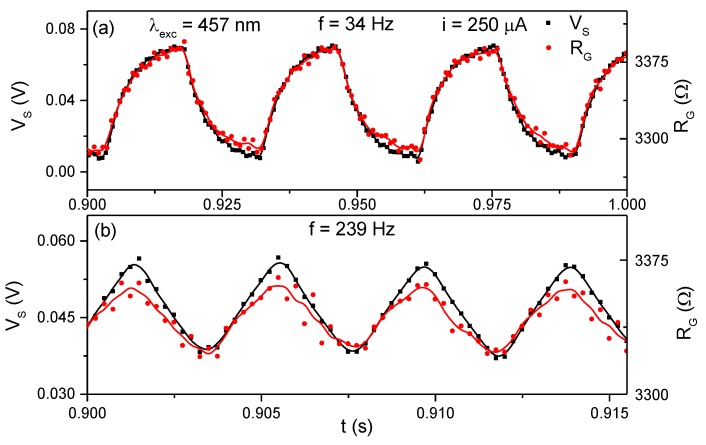
Seebeck open-circuit voltage and the grid resistance measured while the light was chopped at 34 Hz (**a**) and 239 Hz (**b**). The red and black lines are only an eye guide.

To verify the response of the device to oscillating light, an experimental setup with a homemade optical chopper system was used to measure the Seebeck voltage and the grid resistance, R_G_, while the light beam was chopped from 34 Hz to 239 Hz. The measurements were performed in air at atmospheric pressure. An Ar^+^ laser was used as a light source at 457 nm, operating at 5 mW. Figure 5 shows the Seebeck voltage and the grid resistance measurements carried out at 34 Hz and 239 Hz. The Seebeck voltage and the grid resistance oscillated as a function of the chopping frequency due to the temperature differences caused by the laser light. Both V_S_ and R_G_ presented the same behavior at the several frequencies used in this experiment. Furthermore, it was verified that the sensor can measure oscillations of light until at least 239 Hz. This value was limited by our experimental setup, therefore it is expected that the device will be able to efficiently measure variations in a higher frequency range. By fitting the voltage curve in Figure 5a, it was also possible to determine the response time of the device, which was ~4.3 ms. The detectivity was evaluated using the equation $D^{*}=R_{S}\cdot \sqrt{A/4kTR}$ [16,17,18], where *R_S_* is the responsivity, *A* the area of the sensing zone, *k* the Boltzmann constant, *T* the temperature and *R* the internal resistance of the device. D* = $2.86\times 10^{7}$ $cmHz^{\left(1/2\right)}W^{-1}$ compared well with other thermoelectric-based CMOS-compatible sensors considering the reduced thermal constant of the device [16]. 

## 4. Conclusions

This study described the use as photosensor of a thermoelectric microsensor based on single-crystalline Si ultrathin films. By decreasing the thickness, the thermal conductance and heat capacity of the Si thermopiles were significantly reduced, therefore substantially improving the thermal insulation of the membrane. The result was an enhancement of the detectivity of the TE device to very small temperature differences. Furthermore, a small thermal time constant was obtained. FEM simulations were used to obtain the real temperature gradients applied to the thermoelectric legs, correcting the non-flat temperature profile generated during the experiments. The linear dependence of the output voltage with the input power facilitates the use of the microsensor as a photosensor. The R_S_ was determined being 13 V/W in air at atmospheric pressure. The measurements performed at fixed power and variable temperature differences demonstrated a slight reduction of the Seebeck coefficient as a result of the photogeneration of electrical carriers by light absorption in the visible range. The measurements carried out with a chopped light beam evidenced the quick response time of the device due to its very small thermal mass, ~4.3 ms. Finally, the D* of the device was $2.86\times 10^{7}$ $cmHz^{\left(1/2\right)}W^{-1}$.

## Supplementary Materials

The following are available online at https://www.mdpi.com/1424-8220/19/6/1427/s1; Figure S1. Transmittance and reflectance spectra of the multilayer stack membrane.

## References

[1] Dennis P.J., Welch E.F., Alarie J.P., Ramsey J.M., Jorgenson J.W. (2010). Development of a Photothermal Absorbance Detector for Use with Microfluidic Devices. Anal. Chem..

[2] Patil V., Capone A., Strauf S., Yang E.-H. (2013). Improved Photoresponse with Enhanced Photoelectric Contribution in Fully Suspended Graphene Photodetectors. Sci. Rep..

[3] García de Arquer F.P., Mihi A., Konstantatos G. (2015). Large-Area Plasmonic-Crystal–Hot-Electron-Based Photodetectors. ACS Photonics.

[4] Chen G.Y., Wu X., Liu X., Lancaster D.G., Monro T.M., Xu H. (2017). Photodetector Based on Vernier-Enhanced Fabry-Perot Interferometers with a Photo-Thermal Coating. Sci. Rep..

[5] Boukai A.I., Bunimovich Y., Tahir-Kheli J., Yu J.-K., Goddard W.A., Heath J.R. (2008). Silicon Nanowires as Efficient Thermoelectric Materials. Nature.

[6] Hochbaum A.I., Chen R., Delgado R.D., Liang W., Garnett E.C., Najarian M., Majumdar A., Yang P. (2008). Enhanced Thermoelectric Performance of Rough Silicon Nanowires. Nature.

[7] Neogi S., Reparaz J.S., Pereira L.F.C., Graczykowski B., Wagner M.R., Sledzinska M., Shchepetov A., Prunnila M., Ahopelto J., Sotomayor-Torres C.M. (2015). Tuning Thermal Transport in Ultrathin Silicon Membranes by Surface Nanoscale Engineering. ACS Nano.

[8] Ferrando-Villalba P., Lopeandia A.F., Abad L., Llobet J., Molina-Ruiz M., Garcia G., Gerbolès M., Alvarez F.X., Goñi A.R., Muñoz-Pascual F.J. (2014). In-Plane Thermal Conductivity of Sub-20 Nm Thick Suspended Mono-Crystalline Si Layers. Nanotechnology.

[9] Li Y., Buddharaju K., Singh N., Lo G.Q., Lee S.J. (2011). Chip-Level Thermoelectric Power Generators Based on High-Density Silicon Nanowire Array Prepared with Top-down CMOS Technology. IEEE Electron Device Lett..

[10] Curtin B.M., Fang E.W., Bowers J.E. (2012). Highly Ordered Vertical Silicon Nanowire Array Composite Thin Films for Thermoelectric Devices. J. Electron. Mater..

[11] Perez-Marín A.P., Lopeandía A.F., Abad L., Ferrando-Villaba P., Garcia G., Lopez A.M., Muñoz-Pascual F.X., Rodríguez-Viejo J. (2014). Micropower Thermoelectric Generator from Thin Si Membranes. Nano Energy.

[12] Haras M., Lacatena V., Morini F., Robillard J.-F., Monfray S., Skotnicki T., Dubois E. (2015). Thermoelectric Energy Conversion: How Good Can Silicon Be?. Mater. Lett..

[13] Li D., Wu Y., Kim P., Shi L., Yang P., Majumdar A. (2003). Thermal Conductivity of Individual Silicon Nanowires. Appl. Phys. Lett..

[14] Cahill D.G., Braun P.V., Chen G., Clarke D.R., Fan S., Goodson K.E., Keblinski P., King W.P., Mahan G.D., Majumdar A. (2014). Nanoscale Thermal Transport. II. 2003–2012. Appl. Phys. Rev..

[15] Malhotra A., Maldovan M. (2016). Impact of Phonon Surface Scattering on Thermal Energy Distribution of Si and SiGe Nanowires. Sci. Rep..

[16] Dillner U., Kessler E., Meyer H.-G. (2013). Figures of Merit of Thermoelectric and Bolometric Thermal Radiation Sensors. J. Sens. Sens. Syst..

[17] Wang K., Xue C., Liang T., Jiao B., Zhang W., Chen D., Xiong J. (2010). Thermopile Infrared Detector with Detectivity Greater Than 108 CmHz(1/2)/W. J. Infrared Millim. Terahertz Waves.

[18] Zhou H., Kropelnicki P., Tsai J.M., Lee C. (2013). Development of a Thermopile Infrared Sensor Using Stacked Double Polycrystalline Silicon Layers Based on the {CMOS} Process. J. Micromech. Microeng..

[19] Harper J.G., Matthews H.E., Bube R.H. (1970). Photothermoelectric Effects in Semiconductors: N- and P-Type Silicon. J. Appl. Phys..

[20] Kwok H., Bube R.H. (1970). Another Look at the Anomalous Photothermoelectric Effect in P-Type Silicon. J. Appl. Phys..

[21] Okazaki R., Horikawa A., Yasui Y., Terasaki I. (2012). Photo-Seebeck Effect in ZnO. J. Phys. Soc. Jpn..

[22] Mondal P.S., Okazaki R., Taniguchi H., Terasaki I. (2013). Photo-Seebeck Effect in Tetragonal PbO Single Crystals. J. Appl. Phys..

[23] Lv Y., Chen J., Zheng R.-K., Song J., Zhang T., Li X., Shi X., Chen L. (2015). Photo-Induced Enhancement of the Power Factor of Cu2S Thermoelectric Films. Sci. Rep..

