# Supplementary material for: Thermoelectric Photosensor Based on Ultrathin Single-Crystalline Si Films
Source: Sensors (Basel). 2019 Mar 22;19(6):1427. doi: 10.3390/s19061427 (PMC6471348; doi:10.3390/s19061427)
Supplement: Supplementary file 1 [file sensors-19-01427-s001.pdf]

Supplementary Materials

Thermoelectric Photosensor Based on Ultrathin Single-Crystalline Si Films †

Gustavo Gonçalves Dalkiranis [1,*], Pablo Ferrando-Villalba [1,‡], Aitor Lopeandia-Fernández [1], Llibertat Abad-Muñoz [2] and Javier Rodríguez-Viejo [1]

[1] Grup de Nanomaterials i Microsistemes, Departament de Física, Universitat Autònoma de Barcelona, Bellaterra, Barcelona 08193, Spain; pablo.ferrandovillalba@imec.be (P.F.-V.); aitor.lopeandia@uab.cat (A.F.-L.); javier.rodriguez@uab.cat (J.R.-V.)

[2] Instituto de Microelectrónica de Barcelona—Centre Nacional de Microelectrònica, Campus UAB, Bellaterra, Barcelona 08193, Spain; llibertat.abad@imb-cnm.csic.es (L.A.-M.)

* Correspondence: dalkiranis@gmail.com; Tel.: +34-93-581-1481

† This paper is an extension version of the conference paper: "Thermoelectric Microsensor Based on Ultrathin Si films"; Gustavo Gonçalves Dalkiranis, Pablo Ferrando-Villalba, Aitor Lopeandía-Fernández, Llibertat Abad-Muñoz, Javier Rodríguez-Viejo in Proceedings of the Eurosensors 2018 Conference, Graz, Austria, 9–12 September 2018.

‡ Present address: IMEC, Kapeldreef 75, 3001 Leuven, Belgium

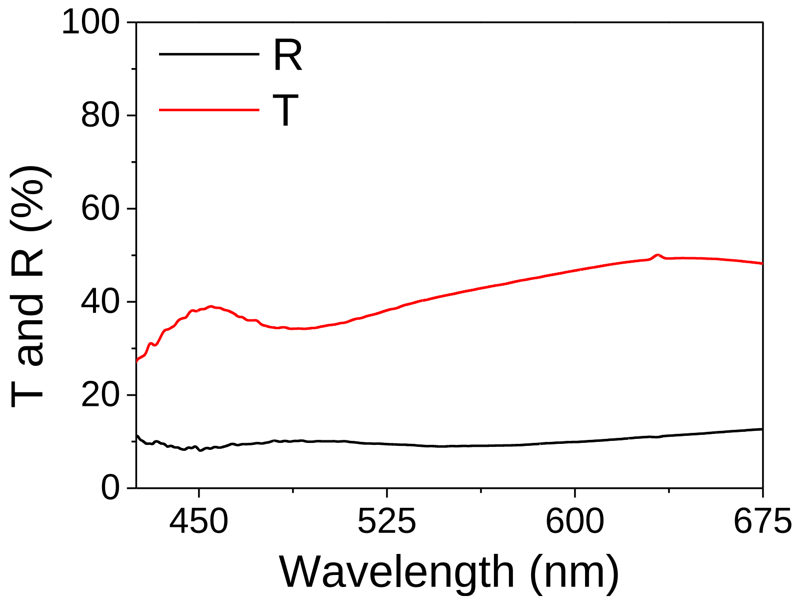

Figure S1: Transmittance and reflectance spectra of the multilayer stack membrane.
